# Sprayable gelatin microparticles prevent delayed gastric bleeding in an anticoagulated swine model

**DOI:** 10.1038/s41598-026-38423-9

**Published:** 2026-02-03

**Authors:** Shohei Uehara, Fumisato Sasaki, Hidehito Maeda, Makoto Hinokuchi, Akihito Tanaka, Shiho Arima, Shinichi Hashimoto, Shuji Kanmura, Hisashi Sahara, Yasuko Kobayashi, Akihiro Nishiguchi, Tetsushi Taguchi

**Affiliations:** 1 Department of Digestive and Life-style related Diseases, Kagoshima University Graduate School of Medical and Dental Sciences, Kagoshima, Japan; 2 Division of Experimental Large Animal Research, Life Science and Laboratory Animal Research Unit, Center for Advanced Science Research and Promotion, Kagoshima University, Kagoshima, Japan; 3 Research Center for Macromolecules and Biomaterials, National Institute for Materials Science, Tsukuba, Japan

**Keywords:** Endoscopic submucosal dissection, Endoscopic mucosal resection, Delayed gastric bleeding, Anticoagulation therapy, Hydrophobized microparticles, animal model, Diseases, Gastroenterology, Medical research

## Abstract

Delayed bleeding following endoscopic mucosal resection or endoscopic submucosal dissection is a serious concern in patients receiving anticoagulant therapy. We previously established an experimental animal model of delayed gastric bleeding following endoscopic resection under controlled anticoagulation. In this study, we aimed to evaluate the potential preventive effect of hydrophobized microparticles (hMPs), a novel sprayable wound-covering material derived from Alaska pollock gelatin, in preventing delayed bleeding. Twelve gastric mucosal defects were created by endoscopic mucosal resection with ligation in three CLAWN miniature swine. To induce delayed bleeding, systemic heparin was administered after confirming an activated clotting time greater than 220 s. hMPs were endoscopically sprayed into each ulcer base (200 mg/ulcer). Endoscopic examination was performed 24 h later. The primary endpoint was the presence or absence of delayed bleeding. Secondary endpoints included hMP retention, ulcer coverage rate, presence of exposed vessels, and vascular coverage by hMPs. During the 24-hour observation period, no delayed bleeding was observed in any of the three CLAWN miniature swine treated with hMPs. Hemoglobin levels on Day 1 remained stable in all swine. hMPs were present in 100% of the ulcers. Complete ulcer base coverage (100%) was achieved in 55.6% (20/36) of the sections, with ≥ 50% coverage observed in all sections. Exposed vessels were identified in 36.1% (13/36) of ulcers, and all were covered with hMPs. Endoscopic spray application of hMPs demonstrated strong adhesion to gastric ulcers and was associated with the absence of delayed bleeding in this short-term experimental model. These findings suggest that hMPs may represent a promising strategy for reducing the risk of delayed bleeding after gastrointestinal endoscopic surgery.

**Supplementary Information:**

The online version contains supplementary material available at 10.1038/s41598-026-38423-9.

## Introduction

Endoscopic submucosal dissection (ESD) and endoscopic mucosal resection (EMR) are well-established treatments for early gastric tumors^1^. Delayed bleeding occurs in approximately 1.8–15.6% of patients after ESD^2,3^, with antithrombotic therapy recognized as a significant risk factor. According to a recently proposed predictive model from Japan, the incidence of delayed bleeding ranges from 2.8% in the low-risk group to 29.7% in the very-high-risk group^4^. With an aging population, the number of patients receiving antithrombotic therapy continues to increase^5^, making delayed bleeding after ESD an increasingly important clinical concern.

Despite its clinical importance, no definitive strategy has been established for preventing delayed bleeding after ESD^6^. A major limitation in developing such interventions is the lack of a reliable animal model for delayed post-ESD bleeding. We recently established such a model using gastric mucosal resection with systemic anticoagulation via a single bolus of heparin followed by continuous infusion^7^. We developed a sprayable wound dressing comprising multifunctional hydrophobized microparticles (hMPs) derived from swine gelatin^7^. These particles demonstrate strong tissue adhesion, even in wet environments, and effectively suppress submucosal fibrosis in the post-ESD setting^7,8^. More recently, we developed hMPs using fine particles derived from Alaska pollock gelatin, which maintained strong adhesive properties^9,10^. These hMPs have shown efficacy in closing gastrointestinal perforations, reducing inflammation in duodenal ESD ulcers^11^, and preventing esophageal strictures in animal models^12^.

In this study, we aimed to evaluate the efficacy of hMPs, a novel sprayable wound-covering material derived from Alaska pollock gelatin, in preventing delayed bleeding.

## Materials and methods

### Experimental animals

Three CLAWN miniature swine (age: 6 months; weight: 14–17 kg; procured from Kagoshima Miniature Swine Research Center, Kagoshima, Japan) were used in this study. As premedication, the animals were intramuscularly injected with ketamine (15 mg/kg; Daiichi Sankyo Propharma Co., Ltd., Tokyo, Japan) or xylazine (2 mg/kg; Bayer Yakuhin, Ltd., Osaka, Japan). An endotracheal tube (Smith Medical Japan, Tokyo, Japan) was inserted, and anesthesia was maintained with inhaled isoflurane (1.5–3.0%) (DS Pharma Animal Health Co., Ltd., Osaka, Japan), delivered via a ventilator, with continuous monitoring of heart rate, respiratory status, and oxygen saturation throughout the procedure.

### Endoscopic mucosal resection with ligation (EMR-L) procedure and post-procedure follow-up

An upper gastrointestinal endoscope was orally inserted into each swine to create gastric mucosal defects using the EMR-L technique^13^. Twelve artificial gastric ulcers were created by an endoscopist by performing the EMR-L technique using an upper gastrointestinal endoscope (GIF-Q260J; Olympus, Tokyo, Japan), and a videoscope system (EVIS LUCERA CV-260SL; Olympus) was used to ensure consistent collection of data on delayed bleeding. Markings were made at 12 locations: lesser curvature, anterior and posterior walls of the upper and middle gastric bodies, gastric angle, and lower gastric body. A polypectomy snare (Captivator™ II 15 mm; Boston Scientific, USA) in coagulation mode was used for marking. One milliliter of 0.9% saline containing indigo carmine (Otsuka Pharmaceutical Co. Ltd., Tokyo, Japan) was injected into the submucosa using an injection needle to achieve adequate lifting. Endoscopic variceal ligation (EVL) was performed using a ligation device (Pneumo Activate EVL; Sumitomo Bakelite, Tokyo, Japan), followed by snaring and resection with a high-frequency device (Pulse-Cut Fast mode, 120 W, ESG-100; Olympus)^13,14^. All EMR-L procedures were performed by S.U. Solid food was withheld from the day before the procedure until the day after; however, water was provided ad libitum. No proton pump inhibitor was administered following EMR-L^7^. Feeding was managed by a specialized animal technician at the Kagoshima University Animal Experimental Facility, and the animals’ conditions were monitored daily. Animal care, housing, and surgical procedures were conducted in compliance with the Kagoshima University Animal Experiment Committee guidelines. Necropsies were performed by several researchers in accordance with the ethical guidelines of the Kagoshima University Animal Experimentation Facility. This study was approved by the Animal Experiment Committee of Kagoshima University (approval number: MD23056).

This study is reported in accordance with the ARRIVE guidelines.

### hMPs

**Fig. 1:**
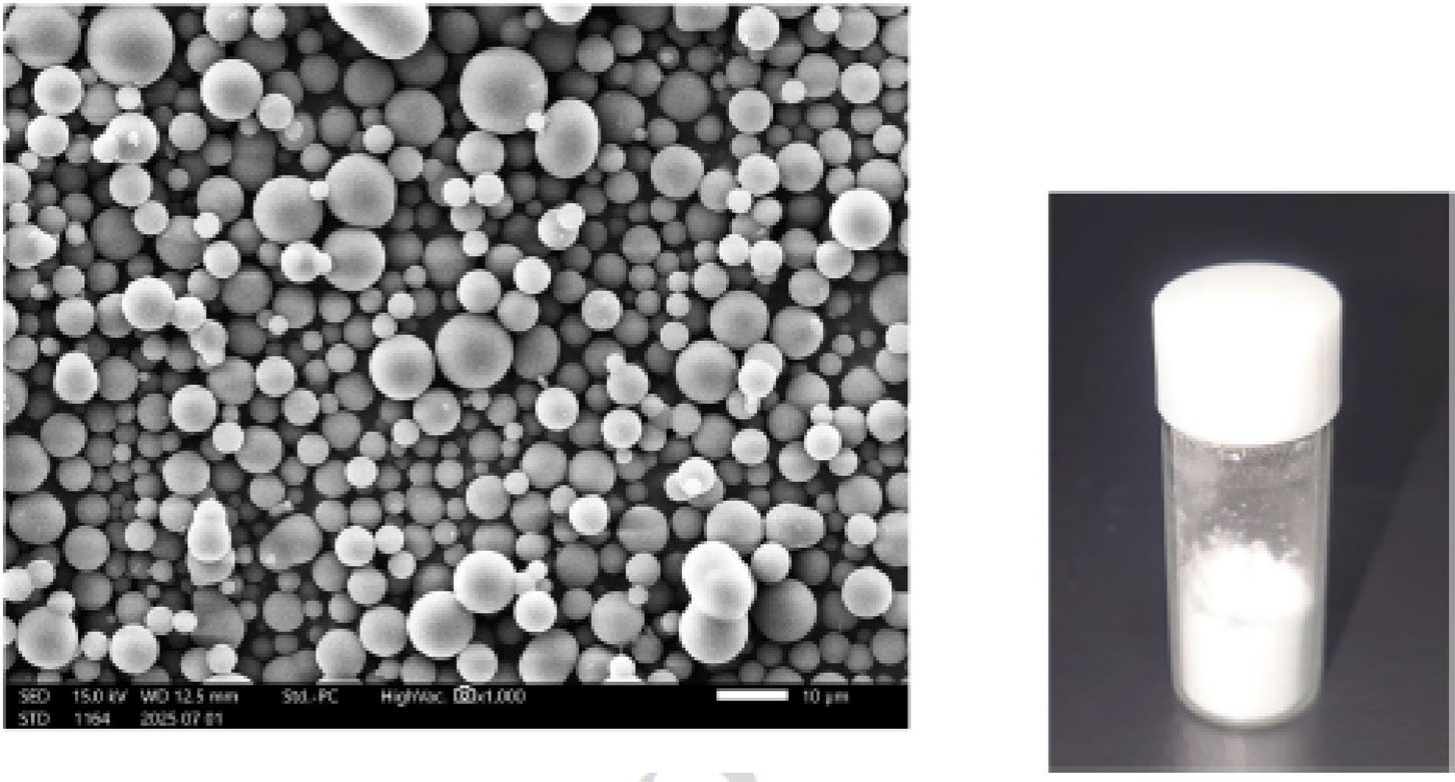
Morphology and appearance of the hMPs Left: SEM image of hMPs shows spherical microparticles with a uniform size distribution (magnification ×2,000) Right: A vial containing dried hMP powder Abbreviations: hMPs, hydrophobized microparticles; SEM, scanning electron microscopy.

Hydrophobically modified Alaska pollock gelatin was synthesized via the reaction between a primary amine and a decanoic anhydrate^15^. The hMPs were prepared using a coacervation method in a water/ethanol mixed solvent^16^. Optimization of the alkyl chain length (decanoyl groups, C10) and the degree of substitution (50 mol% of amino groups in Alaska pollock gelatin) enhanced the mechanical strength of the hydrogel formed by the hydration and fusion of the microparticles. Scanning electron microscopy confirmed that the resulting hMPs were microparticles (Fig. 1).

### Delivery of hMPs

**Fig. 2:**
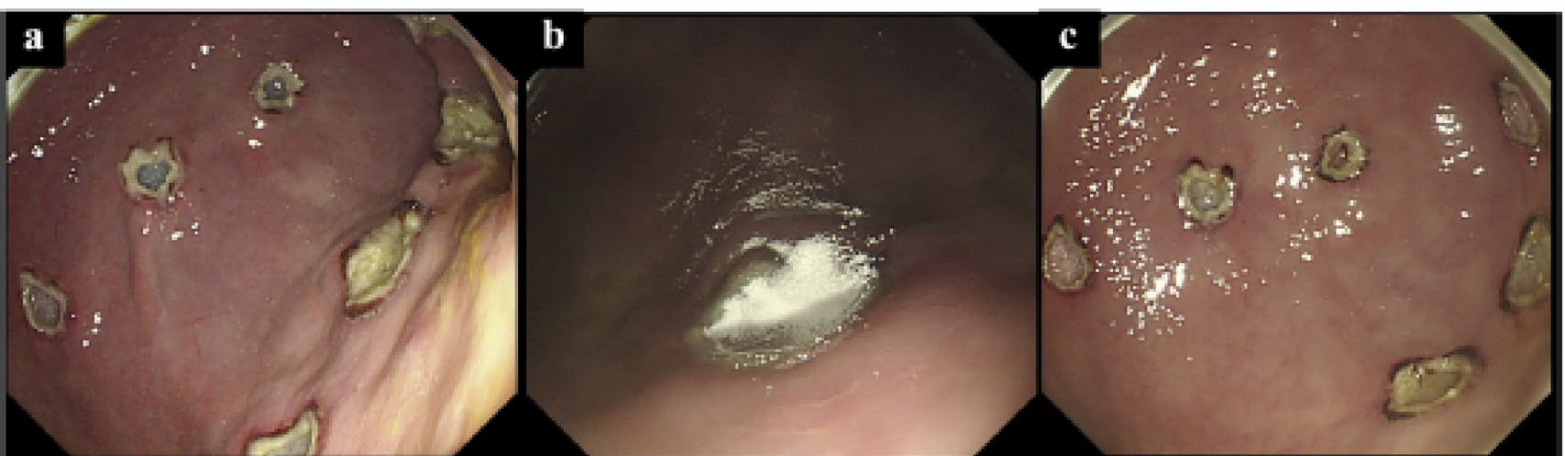
Endoscopic views of hMP application (**a**) Artificial gastric ulcer created via EMR-L (**b**) Appearance immediately after spraying hMPs onto the ulcer base (**c**) Formation of a hydrogel layer as hMPs rapidly gel upon contact with the moist ulcer surface Abbreviations: hMPs, hydrophobized microparticles; EMR-L, endoscopic mucosal resection with ligation.

The hMPs (200 mg) were packed into small vials. A battery-powered endoscopic injector (Alto Shooter^®^, Kaigen, Tokyo, Japan), designed for the application of powdered agents, was used to spray the hMPs. The vial was directly attached to the Alto Shooter^®^, and the powder was sprayed onto the injured mucosa through the endoscope channel after removing the nozzle. Overall, 200 mg hMPs (one vial) were applied to each ulcer (Fig. 2).

### Heparin administration, activated clotting time (ACT) measurement, and swine dissection

**Fig. 3:**
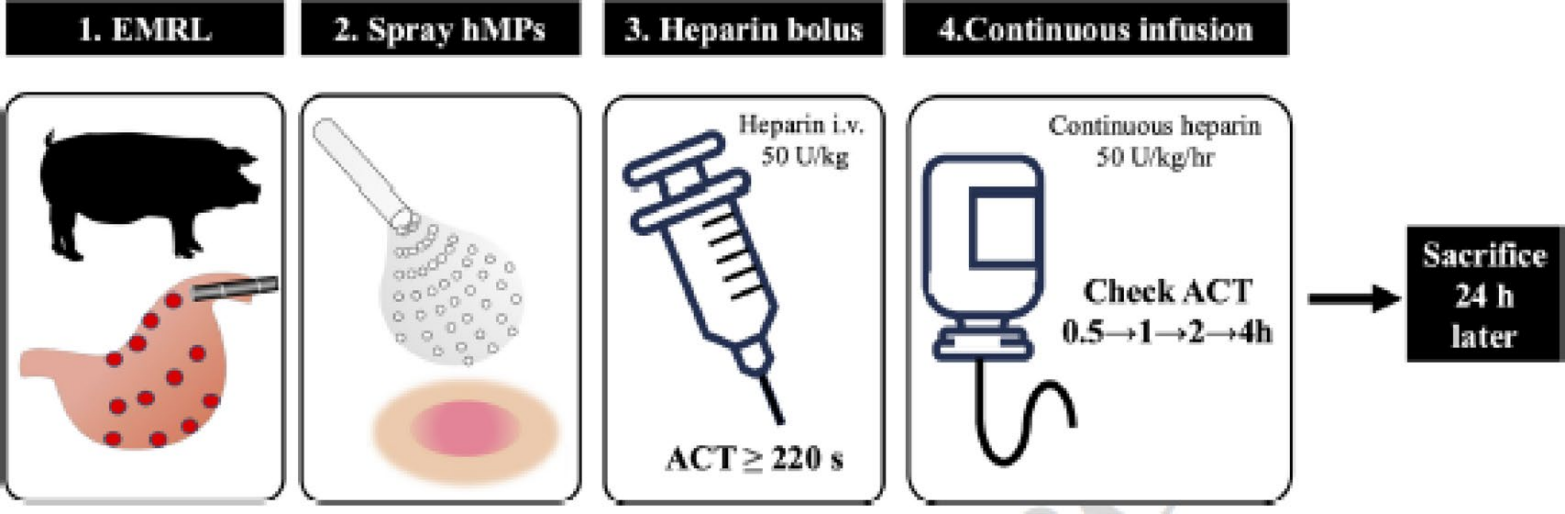
Experimental design of the delayed bleeding model Artificial gastric ulcers are created using EMR-L (step 1), followed by the endoscopic spraying of hMPs on the ulcer base (step 2). A bolus of heparin (50 U/kg) is administered until the ACT exceeds 220 s (step 3). Continuous heparin infusion (50 U/kg/h) is initiated, and ACT is monitored at 0.5, 1, 2, and 4 h (step 4). The animals are sacrificed 24 h after EMR-L, for endoscopic and histological evaluations. Abbreviations: hMPs, hydrophobized microparticles; EMR-L, endoscopic mucosal resection with ligation; ACT, activated clotting time.

All animals received heparin via a catheter inserted into the external jugular vein. Catheters (Argyle Fukuroi; CV catheter, 14 cm × 30 cm; Cardinal Health, USA) were inserted for blood sampling and heparin administration^7^. The experimental protocol is illustrated in Fig. 3. After EMR-L, 50 U/kg unfractionated heparin (heparin sodium; Mochida Pharmaceutical Co., Ltd., Tokyo, Japan) was administered intravenously. The ACT was measured 10 min later using a coagulation analyzer (Hemochron Jr Signature+, Accriva Diagnostics, Inc., USA). Additional doses of 50 U/kg were administered every 10 min until the ACT exceeded 220 s, following a previously established protocol^7^. Continuous heparin infusion (50 U/kg/h) was then initiated using a portable disposable infusion pump (SUREFUSER^®^ A, SFS-1002D, flow rate 2.1 mL/h; NIPRO, Osaka, Japan). ACT was monitored at 0.5, 1, 2, and 4 h after starting the continuous infusion. Endoscopic observation was performed 24 h after EMR-L to assess delayed bleeding. The animals were then euthanized with an intravenous injection of thiamylal sodium (ISOZOL^®^, Nichi-Iko Pharmaceutical Co., Ltd., Toyama, Japan; 90 mg/kg) and potassium chloride (Terumo Corporation, Tokyo, Japan; 20 mEq), followed by abdominal dissection and removal of the stomach.

### Humane endpoints and animal welfare monitoring

All animals were monitored at least twice daily by trained animal technicians for activity level, respiratory pattern, posture, and signs of bleeding. Humane endpoints were predefined as follows: (1) persistent inactivity despite stimulation, (2) signs of respiratory distress, and (3) marked deterioration in physiological status. Animals meeting any of these criteria were humanely euthanized immediately to minimize suffering. No unplanned humane euthanasia was required during the study.

### Histological analyses

Tissue specimens were fixed in 10% neutral-buffered formalin (Kenei Pharmaceutical, Osaka, Japan) for 48 h, sectioned into 20-mm squares, embedded in paraffin, sliced into 2-µm-thick sections, and stained using hematoxylin and eosin or Masson’s trichrome. The retention and coverage of hMPs, the presence of exposed vessels at the ulcer base, and the extent of hMPs adherence to these vessels were assessed histologically.

### Outcome measures

The primary endpoint was the presence or absence of delayed bleeding^7^. Hematemesis was assessed on the day after the procedure, and melena was evaluated during necropsy. Delayed bleeding was diagnosed if at least one of the following criteria was met: (1) hematemesis or vomiting of blood within 24 h after the procedure, or (2) presence of blood clots or retained blood in the stomach observed via endoscopy within 24 h^7^. Hemoglobin (Hb) levels were measured immediately after catheter insertion and again between anesthesia induction and endoscopic observation the next day, and the values were compared.

Secondary endpoints included the retention and coverage rates of hMPs at ulcer sites (*n* = 36), the presence of exposed vessels at the ulcer base, and the proportion of hMPs adherent to those vessels. The coverage rate was defined as the percentage of the ulcer base length covered by hMPs, as assessed on the bisecting plane of the ulcer.

## Results

The characteristics and findings for each swine are presented in Table 1. All 12 ulcers had an approximate diameter of 10 mm. The size of the ulcer was measured using a major forceps. The submucosal layer remained intact at all sites, and the muscularis propria was identifiable. No perforations or active bleeding were observed.

**Table 1:** Characteristics and outcomes.

Model no.	Heparin administration	Continuous heparin dose	ACT at the start of continuous heparin	Max ACT	Vomiting blood	Intragastric blood	Blood clots in ulcers	Hb level before EMR-L	Hb level after EMR-L	Tarry stool	Delayed bleeding
1	+	50 U/kg/h	265	310	-	-	-	11.7	11.7	-	-
2	+	50 U/kg/h	281	347	-	-	-	10.4	10.0	-	-
3	+	50 U/kg/h	236	240	-	-	-	9.2	12.9	-	-

ACT, activated clotting time; Hb, hemoglobin; EMR-L, endoscopic submucosal resection with ligation.

**Fig. 4:**
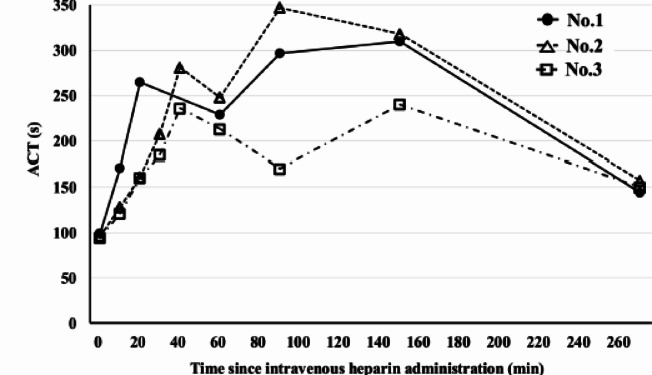
Time course of ACT following intravenous heparin administration Changes in ACT over time in three individual swine (numbers 1–3) are shown. After bolus administration of heparin (50 U/kg), ACT is measured repeatedly, and continuous infusion (50 U/kg/h) is initiated when ACT exceeds 220 s. ACT values are subsequently monitored at multiple time points during the infusion period. Abbreviation: ACT, activated clotting time.

The time course of ACT values following heparin administration is shown in Fig. 4. Heparin (50 U/kg) was repeatedly administered until the ACT exceeded 220 s in all the swine. The ACT values before continuous infusion were 265, 281, and 236 s in the first, second, and third swine, respectively. The maximum recorded ACT values were 310, 347, and 240 s, respectively.

**Fig. 5:**
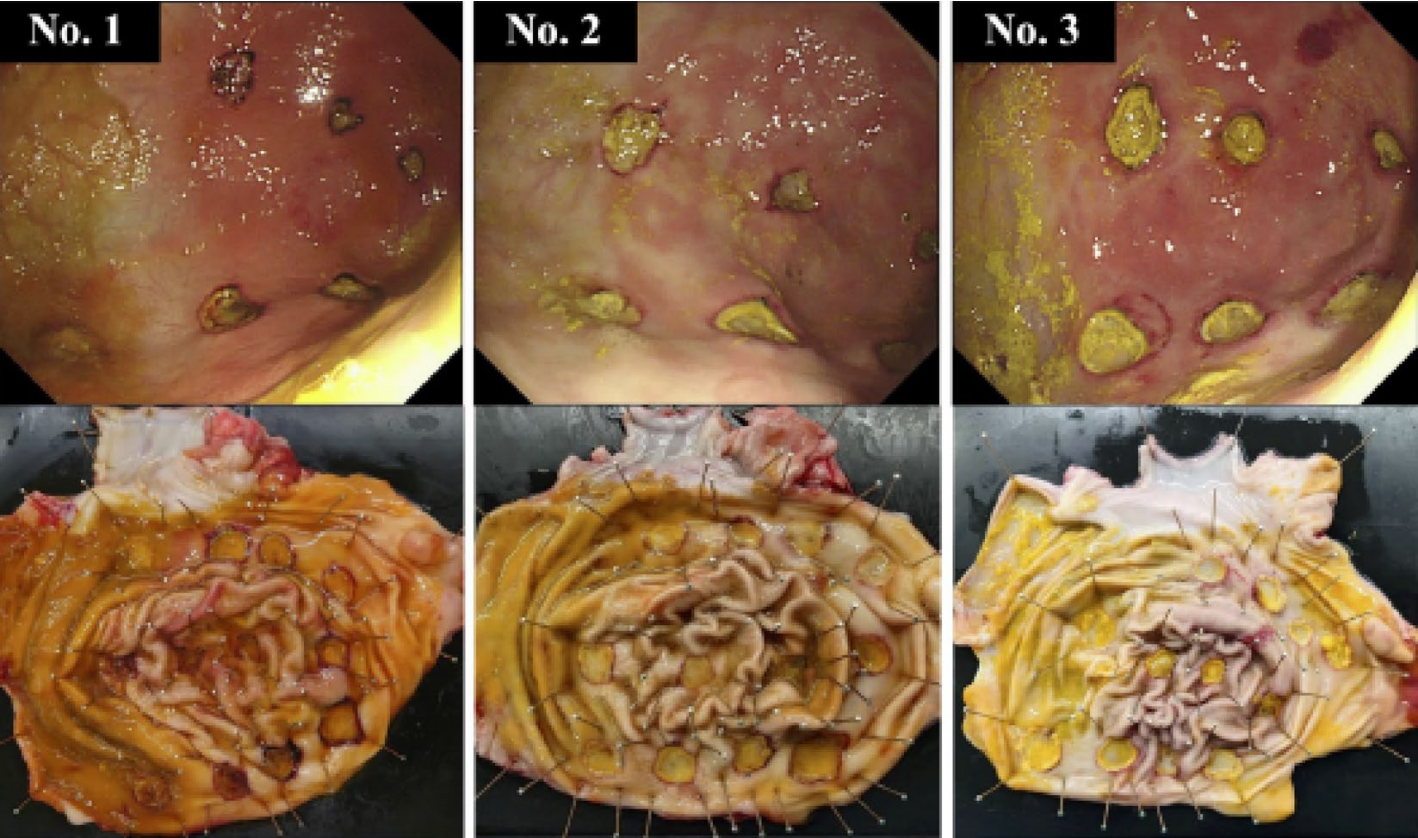
Endoscopic and gross anatomical images of the stomach 24 h after the procedure in three swine. Upper panels: Endoscopic views from swine numbers 1–3 showing ulcer bases covered with hMPs Lower panels: Gross anatomical views of resected stomachs showing no evidence of delayed bleeding (no active bleeding or adherent clots). Abbreviation: hMPs, hydrophobized microparticles.

**Fig. 6:**
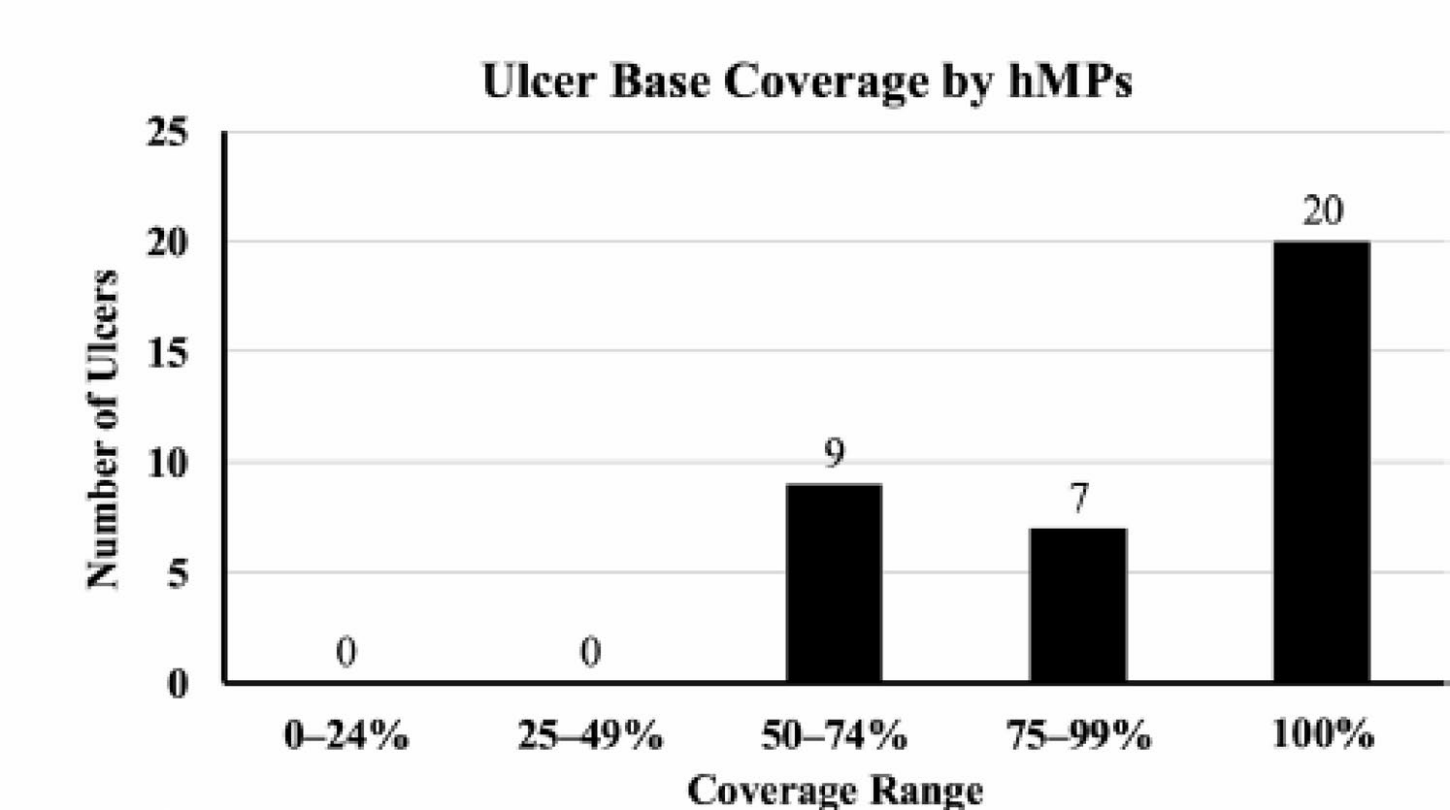
Ulcer base coverage by hMPs Distribution of ulcer base coverage by hMPs in 36 ulcers. Complete coverage (100%) was achieved in 20 ulcers (55.6%), 75–99% coverage in 7 ulcers (19.4%), and 50–74% coverage in 9 ulcers (25.0%). None of the ulcers showed a coverage below 50%. Abbreviation: hMPs, hydrophobized microparticles.

**Fig. 7:**
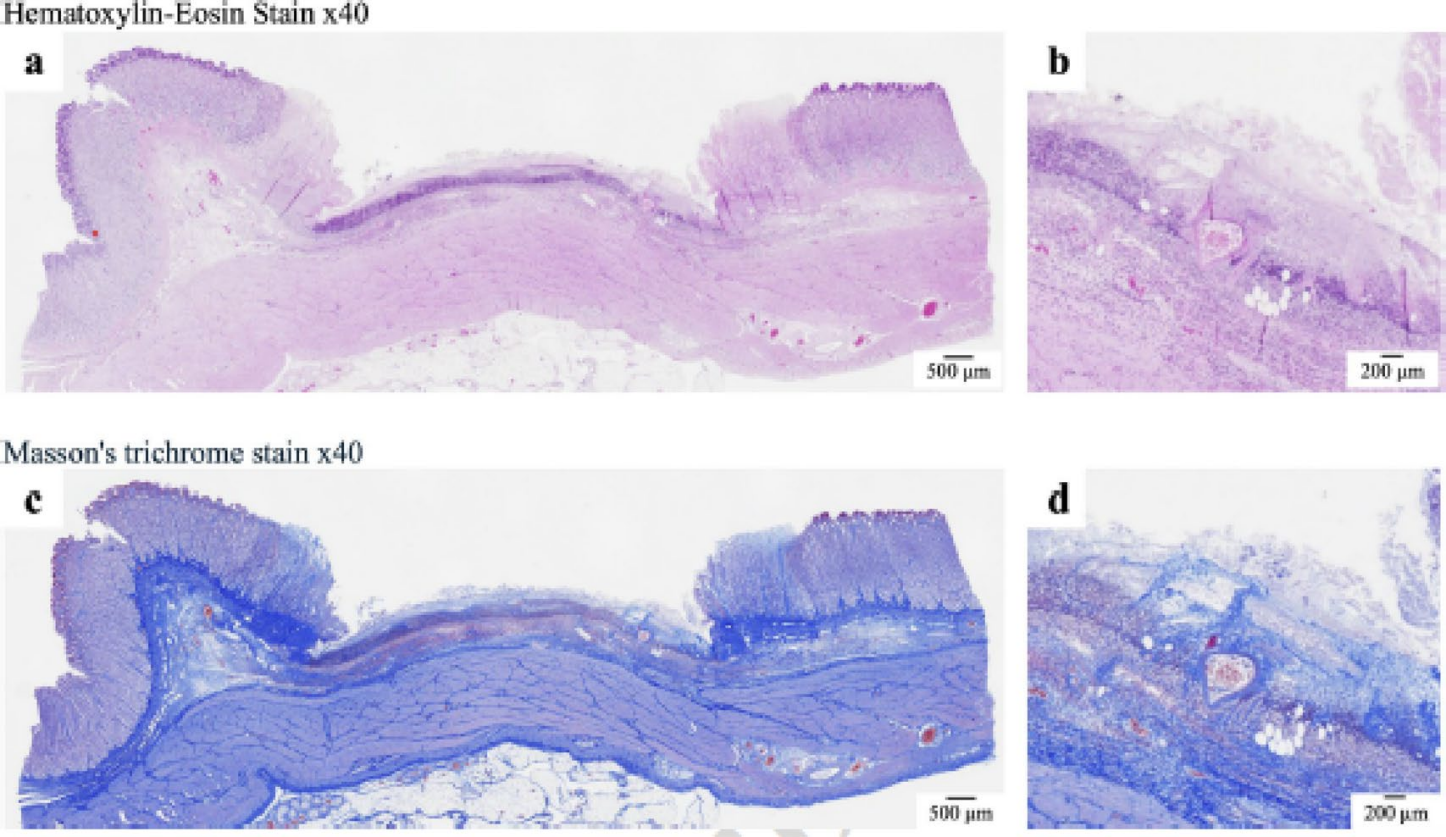
Histological evaluation of ulcer bases after EMR-L and hMP application. The hMPs are retained on the ulcer base following EMR-L and are observed to cover the exposed blood vessels within the submucosa (a, c). Higher-magnification images (b, d) clearly show hMPs adhering to the surfaces of the exposed vessel-like structures.

No delayed bleeding was observed in any swine treated with hMPs (Fig. 5). Hb levels were 10.4 ± 1.0 g/dL at baseline and 11.5 ± 1.2 g/dL after treatment. The mean change was 1.1 ± 1.8 g/dL. The Hb levels remained stable in all swine(Before EMR-L: 10.4 ± 1.0 g/dL, After EMR-L: 11.5 ± 1.2 g/dL). hMPs were detected in all 36 ulcer sections, with a 100% retention rate. Complete coverage (100%) of the ulcer base was observed in 55.6% (20/36); 75–99%, in 19.4% (7/36); and 50–74%, in 25.0% (9/36); all ulcers exhibited ≥ 50% coverage (Fig. 6). Exposed vessels were identified in 36.1% (13/36) of the ulcer bases, and all were covered by hMPs. Representative histological images are shown in Fig. 7. hMPs were firmly attached to the ulcer base and provided additional coverage over the exposed vessels.

## Discussion

In this study, we evaluated the preventive efficacy of hMPs derived from Alaska pollock gelatin using a newly established animal model of delayed bleeding after endoscopic treatment. Under the conditions of this study, no delayed bleeding was observed, and relatively high ulcer coverage rates as well as stable adherence to exposed vessels were confirmed, suggesting that hMPs may contribute to the prevention of delayed bleeding in an anticoagulated swine model.

Delayed bleeding following endoscopic procedures is a serious adverse event in patients receiving antithrombotic therapy^2,3^. Various preventive strategies, including mechanical closure and the use of fibrin sealants, have been investigated^17–20^.The hMPs used in this study demonstrated strong adhesive properties even in wet environments, which is consistent with the previous success of gelatin-based derivatives in ulcer protection and stricture prevention, supporting their potential applicability as a wound-covering material in the gastrointestinal tract.

A notable strength of this study is that it is the first report of a preventive intervention against delayed bleeding in our newly developed^7^, reproducible post-endoscopic bleeding animal model. This model provides a valuable platform for the preclinical evaluation of various hemostatic and wound-covering technologies.

**Fig. 8:**
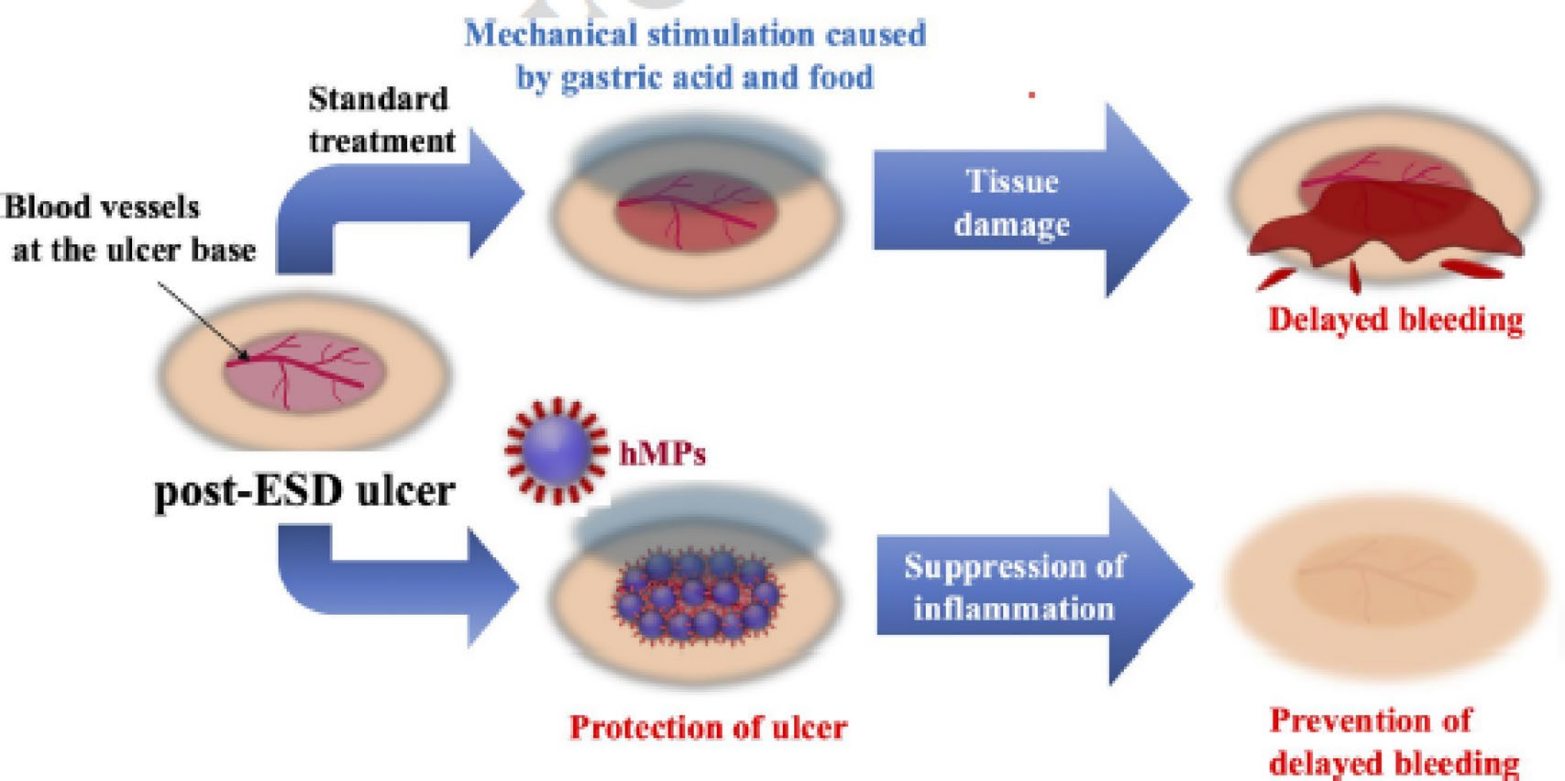
Proposed mechanism of delayed bleeding prevention by hMPs. Post-ESD ulcers are susceptible to damage and delayed bleeding under standard conditions. Spraying hMPs on the ulcer base creates a protective barrier, suppresses local inflammation, and prevents bleeding. hMPs can be easily delivered through an endoscope. Abbreviations: hMPs, hydrophobized microparticles; ESD, endoscopic submucosal dissection.

We hypothesized that hMPs prevent delayed bleeding by firmly adhering to the ulcer base and shielding exposed vessels from chemical and mechanical insults such as gastric acid and food residue. Histological evaluation confirmed that the hMPs adhered to and covered the exposed vessels. Thus, the endoscopic application of hMPs may effectively prevent delayed bleeding following gastric mucosal resection (Fig. 8).

The clinical significance of our findings lies in their potential to offer a novel prophylactic strategy for high-risk patients after gastric ESD, including those receiving anticoagulant therapy. If implemented in clinical practice, this technology could not only reduce the risk of bleeding but also decrease the procedural workload for physicians and offer economic benefits by shortening hospital stays and avoiding readmissions. Given the aging population and the increasing prevalence of antithrombotic medication use, hMPs represent a promising and practical option for clinical implementation. This study has some limitations. First, the observation period was limited to 24 h after EMR-L. Although this duration is relatively short for fully evaluating delayed bleeding, previous clinical studies have reported that a substantial proportion of post-ESD or post-EMR bleeding events occur within the first 24 h, particularly in patients receiving antithrombotic therapy^21,22^.Therefore, evaluation during this early phase remains clinically relevant; however, longer-term observation will be required in future studies. Second, this was a preclinical animal study involving only three CLAWN miniature swine, which limited the statistical power and generalizability of the findings. Therefore, these results should be interpreted with caution. Third, no concurrent control group was included. In a previously established swine model using a similar anticoagulation protocol, delayed bleeding occurred in 75% (3 of 4) of swine when continuous heparin infusion was initiated at an ACT threshold of ≥ 200 s, and in all swine when initiated at an ACT threshold of ≥ 220 s^7^. The present study employed the latter, stricter anticoagulation condition, suggesting that this model represents a high-risk setting for delayed bleeding. Although these findings support the feasibility of the observed preventive effect, direct comparison with a concurrent control group would further strengthen the evidence. Fourth, the in vivo retention time of hMPs could not be fully evaluated because all animals were euthanized 24 h after EMR-L. Although hMPs were present at that time, their long-term persistence remains unclear. In addition, the effects of human gastric peristalsis and the digestive environment on hMPs retention require further investigation. Furthermore, mucosal resection was performed using the EMR-L method rather than the ESD; therefore, its usefulness in preventing post-ESD bleeding remains unknown. However, as EMR-L removes almost the entire submucosal layer, similar to ESD, and multiple areas, it may also be useful for preventing post-ESD bleeding. Finally, the gelatin microparticles used in this study are not yet commercially available, and precise cost evaluation is therefore difficult at this stage. Manufacturing costs and cost-effectiveness compared with existing hemostatic strategies will be important considerations for future clinical translation.

Collectively, these limitations indicate that our work represents an initial proof-of-concept study demonstrating the feasibility and potential efficacy of sprayable hMPs in preventing delayed bleeding. Future studies should ideally include larger sample sizes and, ultimately, multicenter clinical trials to assess safety and efficacy, along with investigations exploring its applicability to other gastrointestinal sites and broader high-risk populations.

Endoscopic application of hMPs on mucosal defects resulted in strong tissue adherence and was associated with the absence of delayed bleeding in this high-risk animal model. These findings suggest that hMPs may represent a promising prophylactic strategy for managing post-endoscopic bleeding, particularly in patients receiving anticoagulant therapy.

## Supplementary Information

Below is the link to the electronic supplementary material.

Supplementary Material 1

## Data Availability

All processed data supporting the findings of this study are included in this published article and its supplementary information files. The raw image data are not publicly available due to file size limitations, but are available from the corresponding author upon reasonable request.

## Abbreviations

hMPs: hydrophobized microparticles
EMR-L: endoscopic mucosal resection with ligation
